# Procalcitonin levels and bacterial aetiology among COPD patients admitted to the ICU with severe pneumonia: a prospective cohort study

**DOI:** 10.1186/1471-2334-9-157

**Published:** 2009-09-21

**Authors:** Cédric Daubin, Jean-Jacques Parienti, Sabine Fradin, Astrid Vabret, Michel Ramakers, Nicolas Terzi, François Freymuth, Pierre Charbonneau, Damien du Cheyron

**Affiliations:** 1 The Department of Medical Intensive Care, Caen University Hospital, 14033 Caen Cedex, France; 2 The Department of Infectious Diseases and Biostatistics and Clinical Research, Caen University Hospital, 14033 Caen Cedex, France; 3 The Department of Biochemistry, Caen University Hospital, 14033 Caen Cedex, France; 4 The Department of Virology, Caen University Hospital, 14033 Caen Cedex, France

## Abstract

**Background:**

Serum procalcitonin (PCT) is considered useful in predicting the likeliness of developing bacterial infections in emergency setting. In this study, we describe PCT levels overtime and their relationship with bacterial infection in chronic obstructive pulmonary disease (COPD) critically ill patients with pneumonia.

**Methods:**

We conducted a prospective cohort study in an ICU of a University Hospital. All consecutive COPD patients admitted for pneumonia between September 2005 and September 2006 were included. Respiratory samples were tested for the presence of bacteria and viruses. Procalcitonin was sequentially assessed and patients classified according to the probability of the presence of a bacterial infection.

**Results:**

Thirty four patients were included. The PCT levels were assessed in 32/34 patients, median values were: 0.493 μg/L [IQR, 0.131 to 1.471] at the time of admission, 0.724 μg/L [IQR, 0.167 to 2.646] at six hours, and 0.557 μg/L [IQR, 0.123 to 3.4] at 24 hours. The highest PCT (PCTmax) levels were less than 0.1 μg/L in 3/32 (9%) patients and greater than 0.25 μg/L in 22/32 (69%) patients, suggesting low and high probability of bacterial infection, respectively. Fifteen bacteria and five viruses were detected in 15/34 (44%) patients. Bacteria were not detected in patients with PCTmax levels < 0.1 μg/L. In contrast, bacteria were detected in 4/7 (57%) patients estimated unlikely to have a bacterial infection by PCT levels (PCTmax > 0.1 and < 0.25 μg/L).

**Conclusion:**

Based on these results we suggest that a PCT level cut off > 0.1 μg/L may be more appropriate than 0.25 μg/L (previously proposed for non severe lower respiratory tract infection) to predict the probability of a bacterial infection in severe COPD patients with pneumonia. Further studies testing procalcitonin-based antibiotic strategies are needed in COPD patients with severe pneumonia.

## Background

Severe pneumonia is a common cause of acute exacerbations of chronic obstructive pulmonary disease (AECOPD) [1]. In this setting, a prompt initiation of antibiotics is recommended [2,3]. However, bacterial etiology is only found in approximately 50% of cases [1,4]. Other pathogens, such as respiratory viruses, have been reported in severe AECOPD requiring ventilation [5,6] and pneumonic AECOPD [1]. Moreover, in clinical practice, signs and symptoms of bacterial and viral lower respiratory tract infections widely overlap [7].

Procalcitonin (PCT) has been described as a marker of bacterial infection [8] and thus may help physicians to limit inadequate use of antibiotics [9-11]. We previously investigated the use of PCT in patients with AECOPD, without pneumonia, hospitalized in the ICU [12]. In this study, we assessed PCT levels overtime and their relationship with bacterial infection in chronic obstructive pulmonary disease (COPD) patients admitted to the ICU with severe pneumonia. In addition, we examined whether PCT thresholds predicting probability of bacterial infection previously reported [9-11] could be efficient in this specific population.

## Methods

### Patients

We conducted a monocentric prospective cohort study. All consecutive COPD patients with suspected lower respiratory tract infection admitted to the medical intensive care unit of the University Hospital of Caen between September 2005 and September 2006 were assessed for eligibility. Only those with infiltrates present on initial chest radiographs at admission to ICU and suspected for acute community-acquired pneumonia were included.

### Definition

We defined COPD according to the Global Initiative for Chronic Obstructive Lung Disease Guidelines (GOLD) 2005 http://www.goldcopd.com. Pneumonic AECOPD was defined as a new infiltrate on chest radiograph with features of lower respiratory tract infection in COPD patients [1,11,12]. We used the pneumonia severity index (PSI) to estimate severity [13]. A systematic search for bacteria with standard methods and for viruses with sensitive methods (i.e., PCR and RT-PCR methods), as reported elsewhere [6,12,14-16] were performed. Briefly, sputum or tracheal aspirates were bacteriologically processed if less than 1% of the observed field contained squamous epithelial cells and more than 25 neutrophils were observed [17]. Pneumonia was considered bacteriologically confirmed when at least one of the following criteria were present: pathogen concentration greater than 10^5 ^cfu/mL in tracheobronchial aspirations or sputum samples; blood culture positive for a bacterial pathogen in the absence of an extrapulmonary focus [17-19]. In addition, a serological diagnostic for antibodies to Legionella pneumophila was performed by indirect immunofluorescence, associated with a detection of Legionella pneumophila serogroup 1 antigen in urine samples in all patients.

### Study design

The ethical board decided approval was not necessary given the observational nature of the study. Accordingly, no informed consent was obtained from the patients. At baseline, COPD severity, according to GOLD criteria, other comorbidities, and clinical and biological variables were recorded [12]. All patients were treated according to the recommendations of the French Consensus Conference [3] (i.e., an antibiotic treatment is recommended in severe pneumonia and severe exacerbation of COPD patients requiring admission in ICU).

### Measurement of serum procalcitonin

Procalcitonin (PCT) assessment followed standard methods described elsewhere [12]. The circulating levels of PCT were sequentially assessed at ICU admission (PCT-H0), after six hours (PCT-H6), and after twenty fours (PCT-H24) hours in ICU. All blood samples were analyzed at the end of the study period. PCT was measured using a sensitive immunoassay (Kryptor PCT, Brahms, Hennigsdorf, Germany) with a functional assay sensitivity of 0.06 μg/L, about fourfold above mean normal levels [20]. Patients were classified into three groups based on probability of bacterial infection according to the highest procalcitonin level measured (PCTmax). As previously reported [9-11], the groups were: group 1, PCTmax < 0.1 μg/L indicating an a low probability of a bacterial infection; group 2, PCTmax > 0.1 and < 0.25 μg/L indicating an unlikely or possible bacterial infection infection; and group 3, PCTmax > 0.25 μg/L indicating a high probability of bacterial infection.

### Statistical analysis

Quantitative and qualitative data were expressed as means (+/- SD), or median (interquartile range, IQR) and percentage (with their 95% confidence intervals (CI) based on normal approximation), respectively. Categorical variables were compared using the chi-square or Fischer's exact test, as appropriate. Quantitative variables were compared using the Student *t*-test or the Mann-Whitney non parametric test, as appropriate. The level of significance was set at 0.05 and all tests were two-sided. We used EPI-INFO version 6.04 dfr (EPI-INFO, CDC, Atlanta, GA) for data collection, and EPI-INFO and SAS version 9.1 (SAS Institute Inc, Cary, NC) for data analysis.

## Results

### Patients and baseline characteristics

During the study period, 80 COPD patients with suspected lower respiratory tract infection were admitted to the ICU, 34 had pneumonia. Baseline characteristics of the pneumonic AECOPD patients are shown in Table 1. Twenty three patients (68%) had severe or very severe COPD. During the previous 30-day period, antibiotic or oral steroid therapy for exacerbations of COPD was reported by 7 and 8 patients, respectively. Eleven patients had received antibiotics within the 24 hours preceding ICU admission. With the exception of 4 patients, all had a severe pneumonia (PSI class IV and V). All patients required ventilator support: non invasive ventilation (NIV) in 23 (68%) patients and invasive mechanical ventilation in 20 (58%) patients, 9 of whom received invasive mechanical ventilation after NIV failure. All patients received antibiotics and inhaled steroids and 18 (53%) systemic steroids. The mean length of ICU stay was 29 ± 31 days. The mean length of non-invasive ventilation, invasive mechanical ventilation, and ventilation-free days during the ICU stay were 2.85 ± 5.1 days, 17.8 ± 31.1 days, and 2.56 ± 2.34 days, respectively. Fourteen patients developed septic shock, nine during the first hours following ICU admission and five during their ICU stay. Six patients developed ventilator-associated pneumonia. Twenty two patients were discharged from the hospital. Twelve patients died, eight from septic shock, three from COPD-related respiratory failure, and one from malignant bronchospasm complicated with cardiac arrest.

**Table 1:** Baseline characteristics of all patients and according to the maximum procalcitonin levels measured (PCTmax)

Characteristics	All n = 34*	PCT<0.1 n = 3	0.1<PCT<0.25 n = 7	PCT>0.25 n = 22
Age, yr	70 ± 10	71 ± 10	71 ± 10	69 ± 10
Male sex, no.(%)	28(82)	2(66)	6(86)	18(82)
				
SAPS II score	37 [20-50]	27 [23-30]	31 [24-44]	40 [34-62]
LOD score	6 [4-10]	6 [4-10]	6 [411]	6 [4-10]
				
Comorbidities, no (%)				
Current smokers,	11(32)	0	4(57)	7(32)
Chronic alcohol abuse	8(24)	2(66)	1(14)	4(18)
Obesity	12(35)	3(100)	2(29)	5(23)
Coronary artery disease	14(41)	1(33)	3(43)	9(41)
Hypertensive heart disease	17(50)	1(33)	2(29)	12(55)
Congestive heart disease	9(26)	1(33)	0	8(36)
Diabetes mellitus	13(38)	0	3(43)	8(36)
				
Chronic pseudomonas colonisation, no (%)	4(12)	0	2(29)	2(9)
				
Antibiotics in 24 H prior ICU admission^¶^, no (%)	11(32)	0	2(29)	8(36)
				
During the previous 30-day period^¶¶^				
Antibiotics for AECOPD	7(20)	0	2(29)	5(23)
Oral steroid therapy for AECOPD	8(24)	1(33)	3(43)	5(23)
				
Severity of COPD, no (%)				
GOLD stage I (mild)	2(6)	0	0	2(9)
GOLD stage II (moderate)	9(26)	1(33)	0	6(27)
GOLD stage III (sever)	3(9)	0	0	3(14)
GOLD stage IV (very sever)	20(59)	2(66)	7(100)	11(50)
Home oxygen, no (%)	18(53)	2(66)	5(71)	11(50)
Home non-invasive ventilation, no (%)	6(18)	1(33)	2(28)	3(14)
Oral or inhaled steroid, no (%)	19(56)	2(66)	6(85)	10(45)
				
Examination at ICU admission, no (%)				
Dyspnea	34(100)	3(100)	7(100)	22(100)
Cough	11(32)	1(33)	3(43)	7(32)
Sputum	16(47)	2(66)	3(43)	11(50)
Rales	15(44)	1(33)	1(14)	12(55)
Wheezing	16(47)	2(66)	6(86)	7(32)
Body temperature, °C	37.3 ± 1.2	37.0 ± 0.7	36.5 ± 0.7	37.7 ± 1.3
Leucocytes count (×10^9^/L)	13.7 ± 6.4	11.2 ± 0.4	12.5 ± 3.4	14.9 ± 7.4
				
Pneumonia severity index (PSI)				
PSI I, II, III	4(11)	1(33)	2(28)	1(5)
PSI IV	14(42)	1(33)	4(57)	8(36)
PSI V	16(47)	1(33)	1(14)	13(59)

Values are given as No.(%), median [25%-75% interquartile range], or mean ± standard deviation

* Levels of procalcitonin (PCT) were assessed in 32/34 patients. PCT is given in μg/L

^¶ ^One patient receiving antibiotic in 24 h prior ICU was not assessed for PCT levels

^¶¶ ^Patients treated with antibiotic or oral steroid during the previous 30-day period for exacerbation of COPD were exclusive of those receiving antibiotic in 24 h prior ICU

### PCT measurements and clinical correlates

The median [25%-75% IQR] PCT levels were as follows: at admission PCT-H0 was 0.493 μg/L [0.131-1.471], PCT-H6 was 0.724 μg/L [0.167-2.646], and PCT-H24 was 0.557 μg/L [0.123-3.4].

PCT levels were not different in patients who had received antibiotics within the month or 24 hours prior to ICU admission, compared to antibiotic-naive patients (PCT-H0 0.695 μg/L [0.202-1.139] vs 0.470 μg/L [0.088-1.471], *p *= 0.73 and PCT-H0 0.942 μg/L [0.202-22.110] vs 0.438 μg/L [0.088-0.902], *p *= 0.22, respectively). In addition, steroids prior to ICU admission did not influence PCT levels (p = 0.76). PCT-H0 was significantly higher when abnormal breath sounds or rales were present (PCT-H0 1.661 μg/L [0.745-26.83] vs 0.207 μg/L [0.086-0.470], *p *= 0.0005) and when fever > 38°C was present; PCT-H0 1.495 μg/L [0.828-56.48] vs 0.272 μg/L [0.087-0.902], *p *= 0.0.05). No association was found between PCT-H0 levels and the presence or absence of sputum and cough.

The PCTmax was < 0.1 μg/L in 3/32 patients (9%), between 0.1 and 0.25 μg/L in 7/32 patients (22%), and > 0.25 μg/L in 22/32 patients (69%), including 20 patients with PCTmax > 0.5 μg/L. There were no associations between PCTmax levels > 0.25 μg/L and severity of COPD (*p *= 0.21), Simplified Acute Physiology Score type II (SAPSII) (*p *= 0.15), Logistic Organ Dysfonction (LOD) (*p *= 0.29), and PSI (*p *= 0.08).

### PCTmax levels and bacterial findings

**Figure 1:**
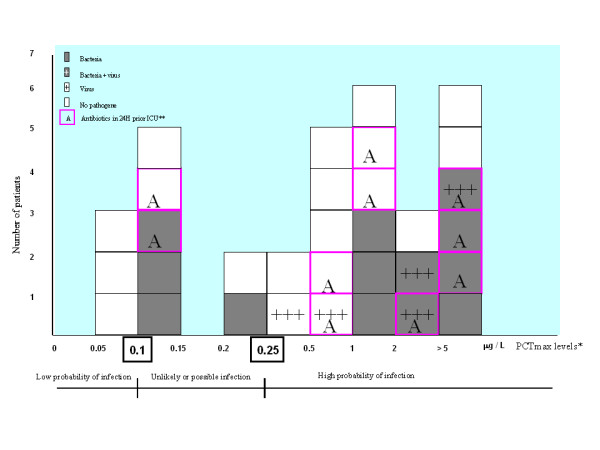
**Distribution of the highest procalcitonin level measurements (PCTmax) and microorganism findings in chronic obstructive pulmonary disease (COPD) patients with pneumonia**. Also shown is the probability of bacterial infection according to procalcitonin (PCT) levels, as previously reported by Christ-Crain (11). * Procalcitonin (PCT) levels were not assessed in 2 patients. ** One patient receiving antibiotic in 24 h prior ICU was not assessed for PCT levels.

**Figure 2:**
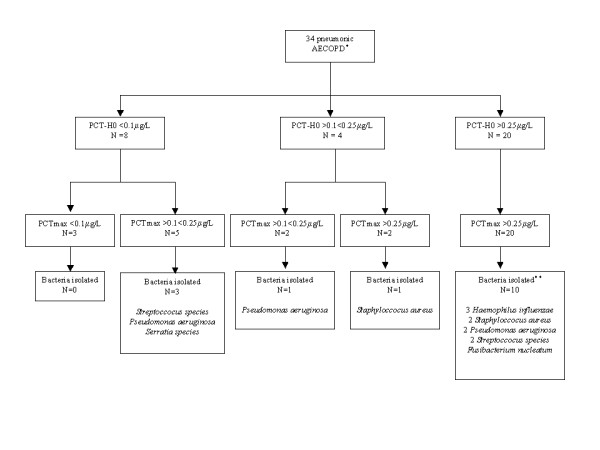
**Dynamics of PCT measurements and bacterial findings**. * Procalcitonin (PCT) levels were not assessed in 2 patients. ** A bacterial co-infection was detected in 2 patients.

Figure 1 depicts bacterial findings according to PCTmax levels. The dynamics of PCT measurements and bacterial findings are shown Figure 2. Fifteen (44%) patients had microbiologically-confirmed pneumonia. Fifteen bacteria (4 *Pseudomonas aeruginosa*, 3 *Haemophilus influenzae*, 3 *Streptoccocus species*, 3 *Methicillin-resistant Staphyloccocus aureus*, 1 *Serratia species*, and 1 *Fusibacterium nucleatum*) and 5 viruses (3 rhinovirus, 1 human metapneumovirus, and 1 respiratory syncitial virus) were detected. A co-infection was detected in 3 cases (rhinovirus and *Streptoccocus*, rhinovirus and *Haemophilus influenzae*, and rhinovirus and *Fusibacterium nucleatum*). Seven patients cross over between PCT groups over time (Figure 2). No bacteria were detected in patients with PCTmax level < 0.1 μg/L. In contrast, bacteria were detected in more than half the patients estimated to have an unlikely bacterial infection (PCTmax > 0.1 and < 0.25 μg/L).

## Discussion

Our study examined microbiological findings and PCT levels in critically ill COPD patients admitted for pneumonia. To our knowledge, this has not been addressed in the specific setting of the ICU. In agreement with previous report [11], PCT level < 0.1 μg/L could indicate a low probability of bacterial infection in approximately 10% of patients (3/32). In contrast, bacteria were detected in more than half the patients (4/7) with PCT levels suggesting an unlikely or possible bacterial infection (PCTmax between 0.1 and 0.25 μg/L), as previously defined outside the ICU [9-11]. Therefore, a PCT cut off below 0.25 μg/L for antibiotic use may not be appropriate for severe pneumonia patients with COPD admitted to the ICU.

We found a median PCT level of 0.493 μg/L [0.131-1.471] in COPD patients admitted to the ICU for pneumonia, this finding is consistent with previous studies focusing on community-acquired pneumonia (CAP) [11,21]. However, in these two large prospective cohorts focusing on CAP, less than 25% of patients had COPD and less than 10% were admitted to the ICU. In previous reports, PCT levels were not different between patients pre-treated with antibiotics and antibiotic-naive patients [10,11], a finding confirmed by our study. In contrast to previous reports, we found an association between PCT levels and clinical symptoms (fever and abnormal breath sounds or rales).

The distribution of patients according to PCTmax levels (ie < 0.1 μg/L, 0.1 to 0.25 μg/L, and > 0.25 μg/L) was similar to that previously reported by Christ-Crain et al [11]. In patients (10%) with PCTmax levels < 0.1 μg/L, bacteria were not detected, suggesting antibiotics may be unnecessary in this subgroup. In contrast, bacteria were detected in more than half the patients with PCT levels suggesting an unlikely or possible bacterial infection (PCTmax > 0.1 and < 0.25 μg/L). This result suggests a PCT level cut-off > 0.1 μg/L, rather than > 0.25 μg/L (proposed for non severe pneumonia [11]), may be more appropriate for initiating antibiotics in a PCT based antibiotic strategy in critically ill COPD patients with pneumonia. Such a study, however, remains to be conducted.

In addition, this study confirms the importance of a sequential PCT levels assessment to predict probability of bacterial infection specifically when the first measurement is low (i.e., PCT < 0.1 μg/L). Indeed, three from five patients crossing over from PCT group < 0.1 μg/L to PCT group 0.1-0.25 μg/L over time, had bacteriologically documented pneumonia. This point should have important implications for furthers studies assessing procalcitonin-based antibiotic strategies in this setting.

We are aware of the limitations of our study, which include the monocentric design and small sample size. In addition, a prior ICU admission antibiotic treatment in a subset of patients may have affected the results of microbiological examinations. However, all patients pre-treated with antibiotics had PCT levels higher than 0.25 μg/L (except two in PCT group 0.1-0.25 μg/L) indicating a high probability of the presence of bacterial infection. Strengths of the study include the dynamic measurement of PCT along with the systematic search for bacteria in a specific population. For this reason, we believe this study adds useful information about PCT levels in COPD patients with severe pneumonia requiring admission to ICU and the likelihood of bacterial infection.

## Conclusion

This study reports that less than 10% of COPD patients suspected of severe pneumonia had PCT levels lower than 0.1 μg/L suggesting a low probability of a bacterial infection and that a subset of patient with PCT levels between 0.1 and 0.25 μg/L (range previously reported as indicating an unlikely or possible bacterial infection [9-11]) had a documented bacterial infection. Based on these results we hypothesize that a PCT level cut off > 0.1 μg/L may be more appropriate than 0.25 (previously proposed for non severe lower respiratory tract infection [9-11] to predict the probability of a bacterial infection in severe COPD patients with pneumonia in a procalcitonin-based antibiotic strategy. However, in clinical practice, a such procalcitonin-based antibiotic strategy using the PCT threshold of 0.1 μg/L should have only a limited impact on the decision to initiate antibiotics in this setting. Further studies are necessary to assess the capability of procalcitonin guidance to shorten antibiotic duration in critically ill COPD patients with pneumonia.

## Abbreviations

AECOPD: acute exacerbation of chronic obstructive pulmonary disease; APACHE II: acute physiology and chronic health evaluation type II; ICU: medical intensive care unit; LOD: logistic organ dysfunction system; PCR: polymerase chain reaction; PSI: pneumonia severity index; SAPS II: simplified acute physiology score II.

## Competing interests

The authors declare that they have no competing interests.

## Authors' contributions

CD and MR initiated the study, the design, and the experimental protocol. SF performed the PCT measurements. AV and FF performed the virologic assessments. CD and JJP performed the statistical analysis and were involved in the interpretation of the results. CD wrote the manuscript, and JJP, MR, and DDC helped to draft the manuscript. DDC, MR, NT, and PC contributed to the conception and design of the study and revision of the manuscript. All authors read and approved the final manuscript.

## Pre-publication history

The pre-publication history for this paper can be accessed here:

http://www.biomedcentral.com/1471-2334/9/157/prepub
